# L-Carnitine Mitigates Oxidative Stress and Disorganization of Cytoskeleton Intermediate Filaments in Cisplatin-Induced Hepato-Renal Toxicity in Rats

**DOI:** 10.3389/fphar.2020.574441

**Published:** 2020-09-29

**Authors:** Ashraf Elkomy, Ehab Yahya Abdelhiee, Sabreen Ezzat Fadl, Mahmoud Abdelghaffar Emam, Fatma Abdel-Monem Gad, Adham Sallam, Saud Alarifi, Mohamed M. Abdel-Daim, Mohamed Aboubakr

**Affiliations:** ^1^ Department of Pharmacology, Faculty of Veterinary Medicine, Benha University, Toukh, Egypt; ^2^ Department of Forensic Medicine and Toxicology, Faculty of Veterinary Medicine, Matrouh University, Matrouh, Egypt; ^3^ Department of Biochemistry, Faculty of Veterinary Medicine, Matrouh University, Matrouh, Egypt; ^4^ Department of Histology, Faculty of Veterinary Medicine, Benha University, Toukh, Egypt; ^5^ Department of Clinical Pathology, Faculty of Veterinary Medicine, Benha University, Toukh, Egypt; ^6^ Department of Zoology, College of Science, King Saud University, Riyadh, Saudi Arabia; ^7^ Department of Pharmacology, Faculty of Veterinary Medicine, Suez Canal University, Ismailia, Egypt

**Keywords:** cisplatin, L-carnitine, hepato-renal toxicity, oxidative stress, intermediate filaments

## Abstract

Cisplatin (CP) is one of the most active medications in cancer treatment and has some adverse effects such as hepatotoxicity and nephrotoxicity. The present research was planned to determine the protective effects of L-carnitine (LC) against CP-induced hepato-renal oxidative stress in rats, *via* investigating of some serum biochemical and tissue oxidative/antioxidant parameters, histological alterations, and immunohistochemical expressions of two different intermediate filaments (IFs) proteins; vimentin (VIM) and cytokeratin 18 (CK18). Twenty-eight rats were divided into four groups (7 rats each). Groups I and II were orally administered saline and LC (100 mg/kg body weight), respectively, once daily for 30 consecutive days. Group III received saline orally once daily and a single dose of CP on the 27th day of the experiment [7.5 mg/kg, intraperitoneal (IP)]. Group IV received both LC and CP. Injection of CP significantly (*P* ≤ 0.05) increased serum alanine aminotransferase (ALT), aspartate aminotransferase (AST), and alkaline phosphatase (ALP) activities and creatinine and urea levels, while serum total protein, albumin, and globulin concentrations significantly (*P* ≤ 0.05) decreased. In addition, CP induced a dramatic increase in the Malondialdehyde (MDA) level along with a substantial decrease in reduced glutathione (GSH) and catalase (CAT) in the hepato-renal tissues. Histologically, both liver and kidney of the CP treated group revealed marked degenerative changes. Moreover, overexpression of both VIM and CK18 in hepato-renal tissues were noted after CP injection. On the other hand, the administration of LC in the CP injected group (Group IV) restored the biochemical parameters, histological, and immunohistochemical pictures toward the normalcy. In conclusion, LC may be supplemented for chemotherapy with CP to ameliorate its oxidative stress and restore the normal organization of IFs, especially VIM and CK18 within the CP intoxicated hepato-renal cells.

## Introduction

Under normal conditions, the various cell types of both liver and kidney have characteristic cytoskeleton and intermediate filaments (IFs) compositions, which are involved in the cell shape maintenance, mechanical stability, and intracellular organization and transport (Ku et al., 1999). Vimentin (VIM) is the IF of the non-epithelial cells, especially those of mesenchymal origin (Sen et al., 2010). The only IF protein found in the endothelial cells and fibroblasts (Evans, 1998). Meanwhile, cytokeratins (CKs) represent the largest and most common epithelial IFs (Snider, 2016). Since chemotherapeutic drugs are metabolized by the liver and excreted through the kidneys, hepato-renal toxicity is a common adverse effect caused by chemotherapy (Abuzinadah and Ahmad, 2020).

Although cisplatin (CP) [*cis*-diamminedichloroplatinum (II), CDDP], is potent anticancer medication use to treat a variety of tumors including testicular, ovarian, bladder, and lung (Ibrahim et al., 2016; Karwasra et al., 2016). Several recent studies recorded that the CP induces hepatotoxicity (Abdellatief et al., 2017; Qu et al., 2019; Abdel-Daim et al., 2020; Hwang et al., 2020) and nephrotoxicity (Abdel-Daim et al., 2017; Abdel-Razek et al., 2020; Abo-Elmaaty et al., 2020; Sadeghi et al., 2020). Miller et al. (2010) reported cisplatin interacts with nuclear and mitochondrial DNA leading to cytotoxicity. Moreover, inflammation, apoptosis, and oxidative stress were mentioned as the most relevant pathways for CP toxicity (Meng et al., 2017) as well as, disorganization of the IFs components of the cytoskeleton (Evans and Simpkins, 1998). In the last decades, VIM is considered a mesenchymal marker for liver and kidney toxicity (Matos et al., 2007; Wang et al., 2017). Moreover, CKs are known as cellular stress protein, specially CK18, which use as novel markers of the liver and kidney injuries (Yang et al., 2015; Djudjaj et al., 2016).

L-carnitine (LC) is a natural nutrient, which synthesis from lysine and methionine essential amino acids. It is derived from dietary sources (75%) and endogenous biosynthesis (25%), mainly in the liver and kidney (Bremer, 1983; Aboubakr et al., 2020). LC is necessary for the production of ATP by β-oxidation of fatty acids in mitochondria (Furuno et al., 2001). Therefore, LC can prevent mitochondrial oxidative stress induces mitochondria damage and apoptosis in different cell types (Barhwal et al., 2007). The major regulatory role of LC in antioxidant processes was discussed in various organs like heart, colon, retina, and brain (Al-Majed et al., 2006; Cetinkaya et al., 2006; Sezen et al., 2008).

From all the above-mentioned data, the purpose of this research was to investigate the protective effects of LC administration on CP-induced hepato-renal injuries in rats *via* investigating some serum biochemical and tissue oxidative/antioxidant parameters. In addition, both histological alterations and immunohistochemical expressions of VIM and CK18 proteins were evaluated in all experimental groups.

## Materials and Methods

### Chemicals

Cisplatin was obtained from (EIMC United Pharmaceuticals, Egypt). Each vial (50 mg/50 ml) was dissolved in physiological saline (0.9% sodium chloride). LC was obtained from MEPACO Company (Inshas Elraml, Egypt). N-ethylmaleimide was obtained from Sigma Al-drich Co., USA. All biochemical analysis kits were purchased from Biodiagnostics Company (Dokki, Giza, Egypt).

### Experimental Animals

The present study was carried out on a total number of 28 white Albino male rats weighed 185 ± 5.7 g. Rats were obtained from the Laboratory Animal Center, Faculty of Veterinary Medicine, Benha University, Egypt. They were acclimatized for two weeks prior to the experiment. The experimental design of the present study was ethically approved by the Ethics Review Committee of the Faculty of Veterinary Medicine, Benha University, Egypt (Approval No. 20181120). All rats received standard laboratory balanced commercial diet and water ad libitum.

### Experimental Design

In the present study, male albino rats were randomly assigned into 4 equal groups (7 rats each). Group I (control), was administered saline orally (the vehicle) once daily for 30 consecutive days. Group II was received LC (100 mg/kg body weight), orally once daily for 30 consecutive days (Avsar et al., 2014). Group III was served as CP toxic control and received saline orally once daily and a single dose of CP on the 27^th^ day of the experiment (7.5 mg/kg, IP; Adeyemi et al., 2017). Group IV was received both LC and CP. The saline or LC was given orally at 10 AM along the experimental period (30 days).

### Sampling

After 24 h from the end of the experiment, rats were anesthetized by isoflurane. Blood samples from each rat were collected by puncturing retro-orbital plexus in sterilized dry centrifuge tubes and kept for 30 min at room temperature (RT) in a slanted position for blood coagulation before centrifugation at 1200 × g for 20 min to obtain serum. The serum was preserved at -20°C until used for biochemical investigations. After blood collection, the animals of all groups were sacrificed by cervical decapitation then the liver and kidneys were excised from each rat and washed with physiological saline. Liver and heart tissues (1 g/sample) were collected in Eppendorf tubes and stored at -80°C until used. A gram specimen of each tissue was homogenized in 5 ml phosphate buffer pH 7.4 using an electrical homogenizer and maintaining the sample on ice. After homogenization, N-ethylmaleimide was added directly to prevent oxidation of GSH. Tissue homogenates were centrifuged at 1,200 × g for 20 min at 4°C. The resulting supernatants were isolated and used in the assessment of the oxidative stress biomarkers in hepatic and renal tissues. Remaining liver and kidney tissues were immediately preserved in 10% neutral buffered formalin for 48 h for histological and immunohistochemical investigations. All rats (28) and remnants of samples were buried in the strict hygienically controlled properly constructed burial pit.

### Serum Biochemical Studies

The activities of alanine aminotransferase (ALT), aspartate aminotransferase (AST) (Reitman and Frankel, 1957), and alkaline phosphatase (ALP) (Tietz et al., 1983) were determined in collected sera as markers for liver injury. While albumin and total protein were determined according to Doumas et al. (1971) and Doumas et al. (1981) as markers for liver function, meanwhile the serum globulin was calculated by the subtraction of albumin from total protein. In addition, the serum levels of urea and creatinine were determined according to Coulombe and Favreau (1963) and Bartels et al. (1972) to evaluate kidney function.

### Detection of Oxidative/Antioxidant Cascades

To detect the oxidative/antioxidant cascades the following measurements were determined, reduced glutathione concentration (GSH; Sedlak and Lindsay, 1968), catalase (CAT; Aebi, 1984) activity, and concentration of malondialdehyde (MDA; Ohkawa et al., 1979) by using special diagnostic kits obtained from Bio diagnostic company, Egypt.

### Histological Examination

Tissue specimens were taken from the liver and kidney of rats in different groups. Then, specimens were dehydrated in serial dilutions of alcohol, cleared in xylene and embedded in paraffin. Paraffin sections of 5 microns thickness were cut and collected on glass slides and stained by hematoxylin and eosin for histological examination (Bancroft et al., 2013).

### Immunohistochemical Studies

Liver and kidney paraffin sections of 5 microns thickness were cut and collected on positively charged slides for immunohistochemical localization of VIM and CK18 using a streptavidin-biotin complex (ABC) method. After, dewaxing, rehydration, and blocking endogenous peroxidase activity, the sections were heated at 90°C with citrate buffer pH 6 for 30 min. Nonspecific staining was blocked 10% bovine serum albumin for 30 min. The sections were then incubated with the primary antibodies (rabbit monoclonal anti-vimentin and anti-cytokeratin 18, Abcam, Boston, the USA at 1:200 dilution) for 1 h at RT. Next, sections were incubated with biotinylated donkey anti-mouse IgG (Abcam, Boston, USA) for 30 min at RT. The visualization of the immunoreactions was observed using a commercial ABC system recommended by the manufacturer (Santa Cruz Biotech, CA, USA). Then the slides were then subjected to diaminobenzene (DAB) as the chromogen and counterstained with hematoxylin. The VIM and CK18 staining in both hepatic and renal tissues of all examined rats were evaluated blindly. At least 5 random high-power fields were checked at a magnification of 400X using Leica DM3000 microscope. Staining features were scored semi-quantitatively according to Liu et al. (2007) as follow: negative (-), weak (+), moderate (++), strong (+++) for no stain, <10% positive cells, 10–50% positive cells, and >50% positive cells, respectively.

### Statistical Analysis

Statistical analysis was performed using SPSS (Version 20.0; SPSS Inc., Chicago, IL, USA). The significant differences between groups were evaluated by one-way ANOVA using the Duncan test as a *post hoc*. Results were expressed as mean ± SEM. P <0.05 was considered significant.

## Results

### Serum Biochemical Analysis

Cisplatin injection significantly (*P* ≤ 0.05) increased serum ALT, AST, and ALP activities compared with those in control rats. Similarly, CP significantly (*P* ≤ 0.05) increased the levels of creatinine and urea. Conversely, serum total protein, albumin, and globulin were significantly (*P* ≤ 0.05) decreased due to CP injection compared to that in control rats. LC administration with CP restored these parameters towards the normal values (**Table 1**).

**Table 1:** Effect of CP and/or LC treatment on serum biochemical parameters in rats (n = 7).

Parameters	Control	LC	CP	LC+ CP
ALT (U/L)	76.36 ± 2.96^c^	69.74 ± 1.95^c^	192.46 ± 2.58^a^	115.57 ± 3.85^b^
AST (U/L)	48.19 ± 4.39^c^	39.55 ± 2.89^c^	107.64 ± 3.72^a^	61.63 ± 5.39^b^
ALP (U/L) T. protein (g/dl)	122.97 ± 3.91^c^ 8.03 ± 0.06^a^	114.87 ± 3.56^c^ 7.59 ± 0.20^ab^	252.01 ± 8.49^a^ 5.29 ± 0.04^c^	184.60 ± 3.53^b^ 7.17 ± 0.28^b^
Albumin (g/dl)	4.48 ± 0.05^a^	4.35 ± 0.11^a^	3.14 ± 0.04^c^	3.78 ± 0.03^b^
Globulin (g/dl)	3.54 ± 0.10^a^	3.23 ± 0.18^a^	2.15 ± 0.04^b^	3.38 ± 0.28^a^
Creatinine (mg/dl)	0.64 ± 0.01^c^	0.59 ± 0.006^c^	1.22 ± 0.05^a^	0.88 ± 0.01^b^
Urea (mg/dl)	32.55 ± 1.14^c^	29.64 ± 1.07^c^	78.03 ± 2.99^a^	54.37 ± 1.98^b^

LC, L carnitine at dose of 100 mg/Kg PO; CP, cisplatin at dose of 7.5 mg/Kg IP; AST, aspartate aminotransferase; ALT, alanine aminotransferase; ALP, alkaline phosphatase; T. protein, total protein.

Data are expressed as the mean ± SE. Within each row, mean with different letters are significantly different at p < 0.05.

### Hepatic and Renal Oxidative Damage Parameters

In the present study, there was substantially increased in MDA level along with a dramatic decrease in GSH and CAT in the liver and kidney tissues of CP-intoxicated rats. Meanwhile, LC+CP administrated group revealed a decrease in MDA level along with elevations in GSH and CAT in hepatic and renal tissues compared with CP treated group (**Table 2**).

**Table 2:** Effect of CP and/or LC treatment on oxidative stress markers in liver and kidney tissues in rats (n = 7).

Parameters	Organ	Control	LC	CP	LC+CP
MDA (nmol/g)	Liver	47.26 ± 1.13^c^	44.29 ± 0.88^c^	116.34 ± 3.17^a^	81.23 ± 3.37^b^
GSH (mg/g) CAT (U/g)	Liver Liver	69.28 ± 2.09^a^ 2.06 ± 0.02^a^	64.25 ± 1.86^a^ 1.98 ± 0.04^a^	34.81 ± 2.25^c^ 1.06 ± 0.04^c^	54.34 ± 2.17^b^ 1.52 ± 0.03^b^
MDA (nmol/g)	Kidney	78.06 ± 2.37^c^	73.19 ± 5.29^c^	182.06 ± 5.16^a^	121.53 ± 4.30^b^
GSH (mg/g)	Kidney	108.98 ± 4.98^a^	101.61 ± 3.47^a^	58.95 ± 3.07^c^	78.65 ± 1.85^b^
CAT (U/g)	Kidney	2.43 ± 0.05^a^	2.22 ± 0.06^b^	1.06 ± 0.04^d^	1.69 ± 0.04^c^

LC, L carnitine at dose of 100 mg/Kg PO; CP, cisplatin at dose of 7.5 mg/Kg IP; MDA, malondialdehyde; GSH, reduced glutathione; CAT, catalase.

Data are expressed as the mean ± SE. Within each row, mean with different letters are significantly different at p < 0.05.

### Histological Observations

**Figure 1:**
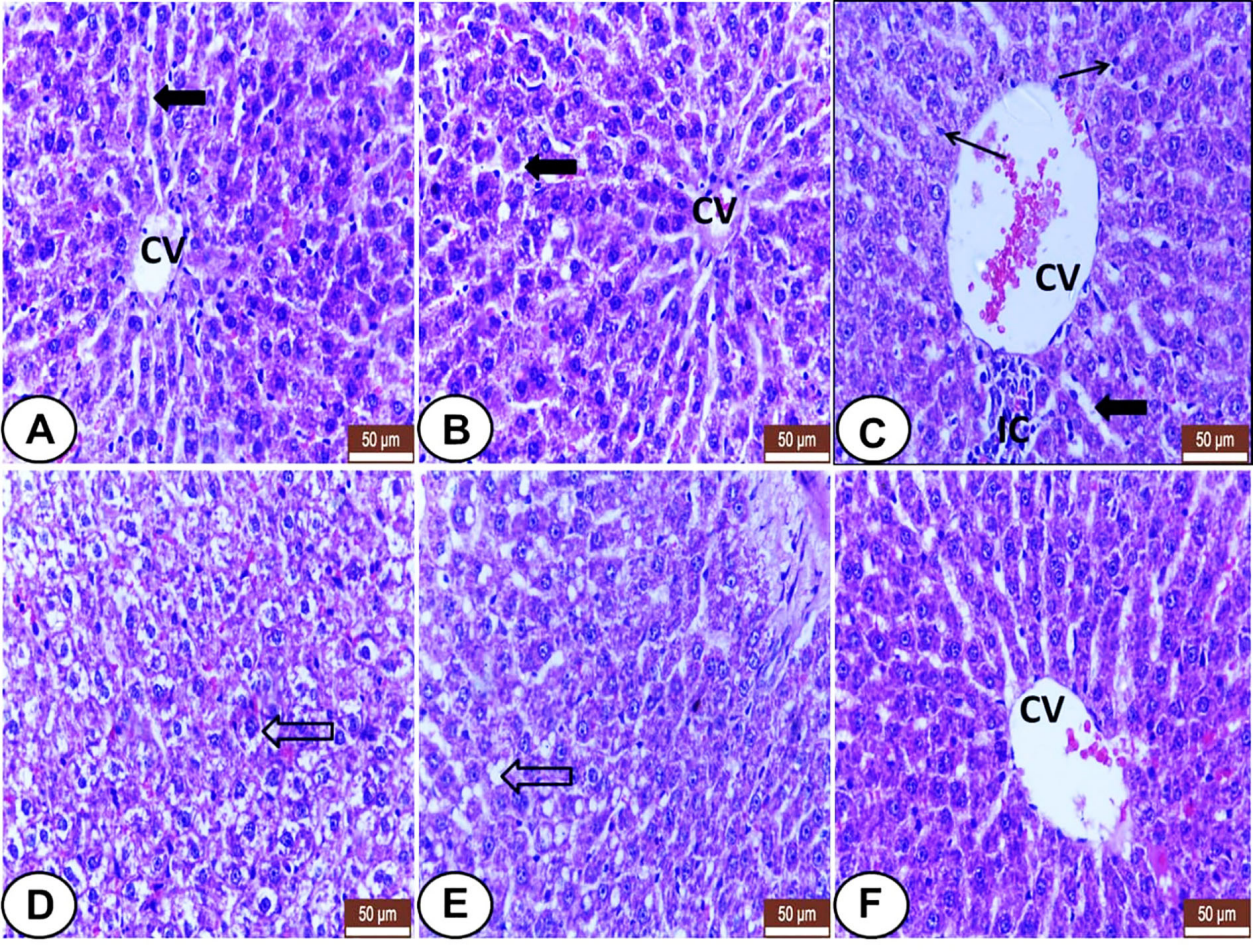
Histological sections of livers from all examined groups. **(A, B)** Control and LC groups showed normal hepatic histo-architecture. Hepatocytes organized in cords radiating from central vein (CV) and separated by regular sinusoids (wide black arrow). **(C–E)** CP treated rats showed several histological changes. **(C)** showed dilatation of the central vein (CV) and sinusoids (wide black arrow), inflammatory cells aggregation (IC), and kupffer cells proliferation (thin arrow). **(D)** showed swelling of hepatocytes, hydropic degeneration (hollow arrow). **(E)** showed fatty infiltration with signet ring appearance in some hepatocytes (hollow arrow). **(F)** LC+CP treated rats showed almost normal hepatocytes and sinusoids, but congested central vein (CV), and no signs of fatty changes were noted. H&E stain, scale bars = 50 µm.

Liver sections from control and LC treated rats exhibited normal hepatic histo-architecture. Hepatocytes organize in cords radiating from the central vein and separate by regular sinusoids (**Figures 1A, B**). Otherwise, CP treated rats revealed several histological changes like dilatation of the central vein and sinusoids, inflammatory cells aggregation, Kupffer cells proliferation (**Figure 1C**), swelling of hepatocytes, hydropic degeneration (**Figure 1D**), and fatty infiltration with signet ring appearance in some hepatocytes (**Figure 1E**). The liver section from LC+CP treated rats represented almost normal hepatocytes and sinusoids, but mild congested central vein and no signs of fatty changes were noted (**Figure 1F**).

**Figure 2:**
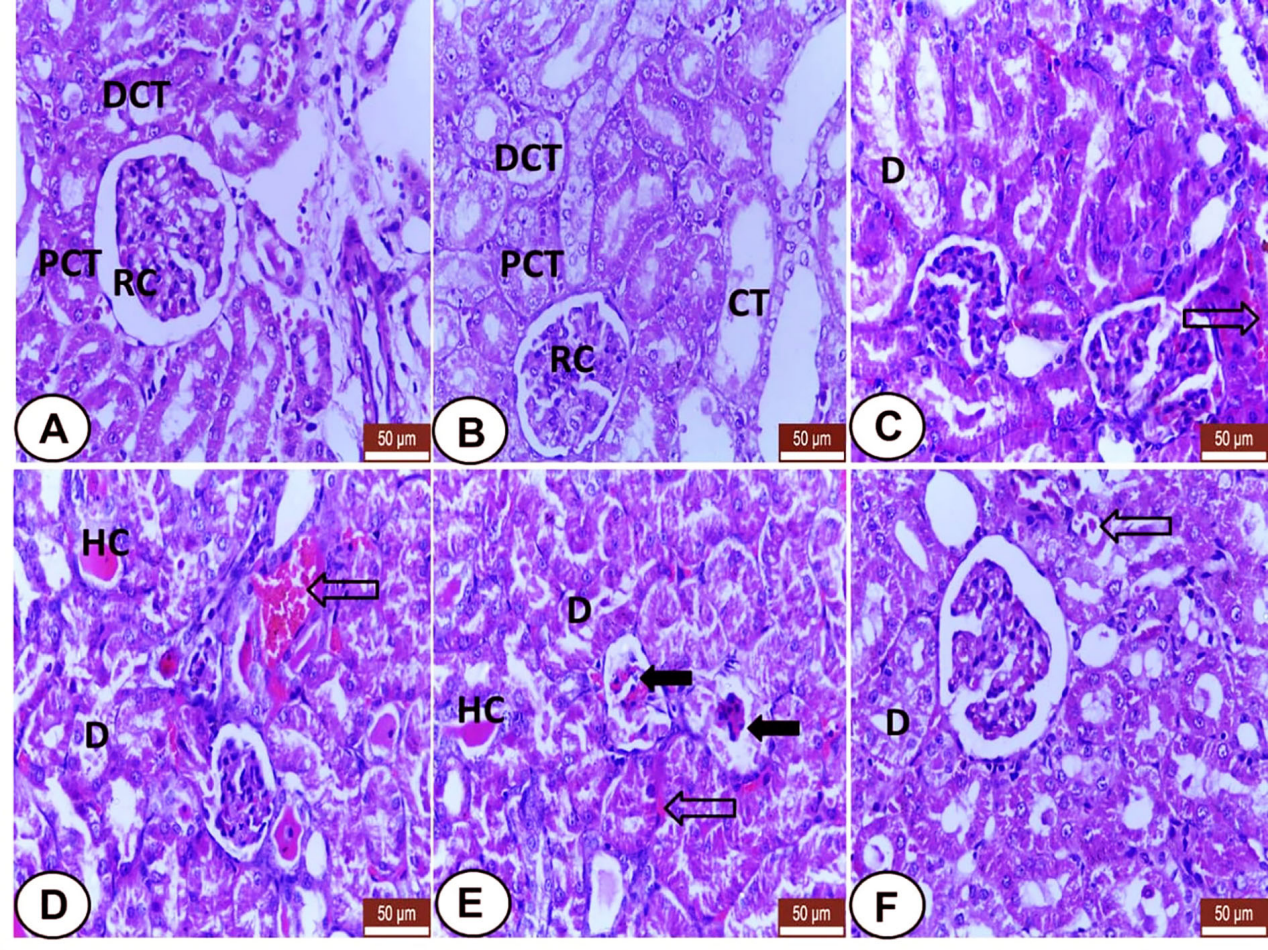
Histological section of kidneys from all examined groups. **(A, B)** Control and LC groups showed regular renal histo-architecture with normal renal corpuscles (RC) and renal tubules; proximal (PCT) distal convoluted tubules (DCT) and collecting tubules (CT). **(C–E)** CP treated rats showed several histological changes. Degenerative changes and desquamation of the tubular epithelia **(D)**, eosinophilic hyaline casts (HC) in some tubules, deformity of some golumeruli with widening of glomerular space (wide black arrows) and congestion of peritubular blood vessels and capillaries (hollow arrows). **(F)** LC+CP treated rats showed mild degenerated tubular epithelium **(D)** with minimal interstitial congestion (hollow arrow). H&E stain, scale bars = 50 µm.

Kidney sections from both control and LC groups showed regular renal histo-architecture with normal renal corpuscles and renal tubules; proximal (PCT) and distal convoluted tubules (DCT) and collecting (CT) tubules (**Figures 2A, B**). In the CP group, many distinguishing histological changes were noted including excessive degenerative changes and desquamation of the tubular epithelia were observed (**Figures 2C–E**) with the presence of eosinophilic hyaline casts in some tubules (**Figures 2D, E**). Also, deformity of some glomeruli with the widening of glomerular space was identified (**Figure 2E**) as well as congestion of peritubular blood vessels and capillaries (**Figures 2C–E**). However, the kidney from the CP+LC group revealed mild tubular degeneration with minimal interstitial congestion (**Figure 2F**).

### Immunohistochemical Observations

A summary of VIM and CK18 immunohistochemical expressions in the livers and kidneys of all examined groups was recorded (**Tables 3**, **4**).

**Table 3:** Summary of VIM and CK18 immunohistochemical expressions in the livers of all examined groups.

Hepatic tissues	VIM				CK18			
	Control	LC	CP	LC+CP	Control	LC	CP	LC+CP
Hepatocytes	–	–	+	–	+	+	+++	++
Sinusoids	+/++	+/++	+++	++	–	–	–	–
Kupffer cells	+/++	+/++	+++	+	–	–	–	–

-, negative; +, weak; ++, moderate; +++, strong.

**Table 4:** Summary of VIM and CK18 immunohistochemical expressions in the kidneys of all examined groups.

Renal tissues	VIM				CK18			
	Control	LC	CP	LC+CP	Control	LC	CP	LC+CP
Renal corpuscles	++	++	++	++	+	+	–	–
Renal tubules	–	–	+++	+	++	++	+++	+
Interstitium	+	+	+++	++	–	–	–	–

-, negative; +, weak; ++, moderate; +++, strong.

### VIM Expression

**Figure 3:**
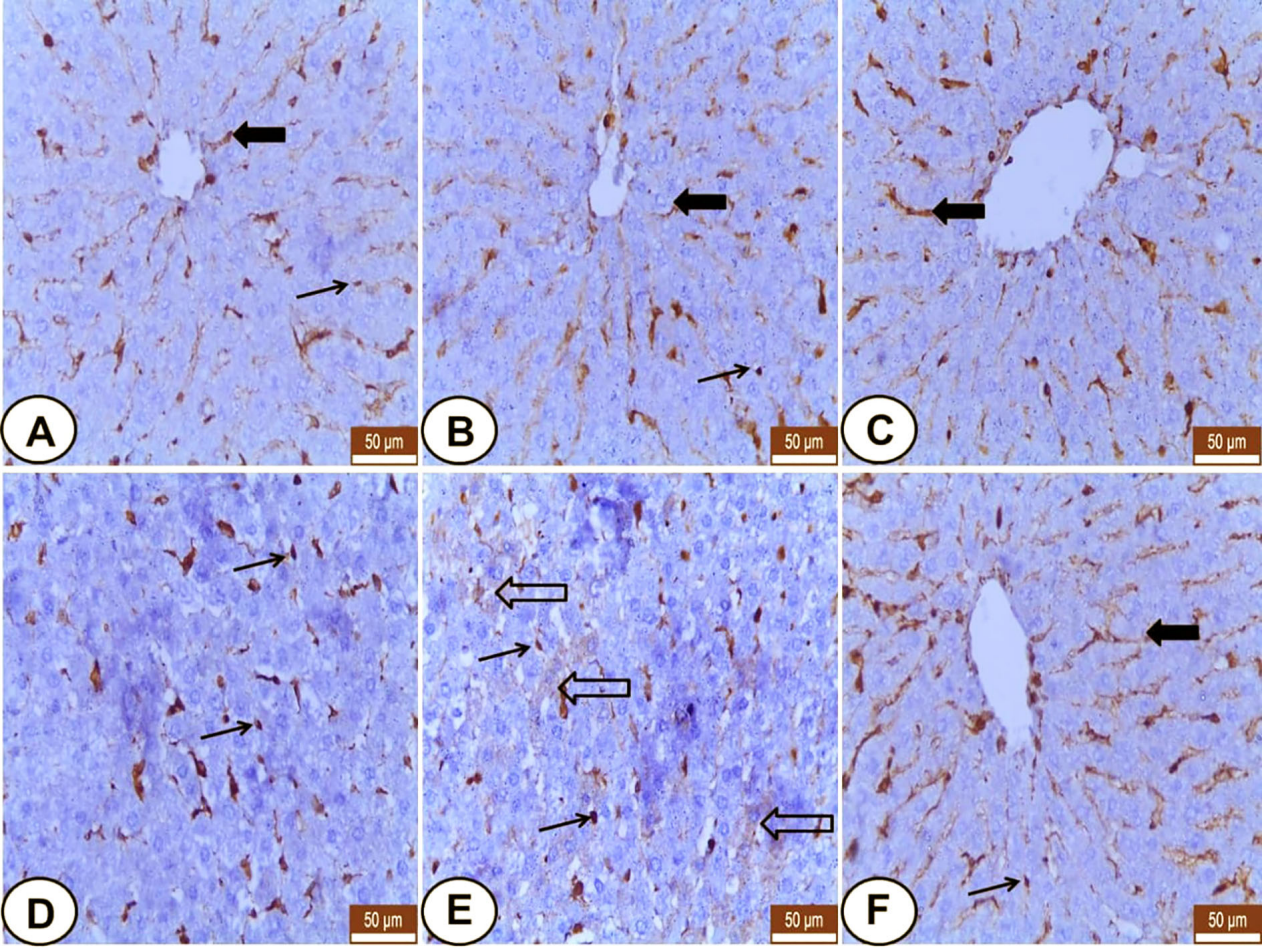
Immunohistochemical staining of VIM in hepatic sections from all examined groups. **(A, B)** Control and LC groups showed weak to moderate hepatic sinusoids (wide arrow) and Kupffer cells (thin arrow) for VIM. **(C–E)** CP group revealed overexpression of VIM in blood sinusoids (wide arrow), Kupffer cells (thin arrows) and some hepatocytes showed weak VIM (hollow arrow). **(F)** LC+CP treated rats showed moderate expression of sinusoidal VIM (wide arrow) and fewer Kupffer cells (thin arrow) compared with CP group. Scale bars = 50 µm.

Both control and LC rats expressed weakly to moderate VIM mainly in the hepatic sinusoids and Kupffer cells, but the hepatocytes were VIM negative (**Figures 3A, B**). Meanwhile, CP-injected rats showed overexpression of VIM in the blood sinusoids (**Figure 3C**) and an increased number of Kupffer cells (**Figures 3D, E**) as well as some hepatocytes labeled weak VIM (**Figure 3E**). The LC+CP group showed moderate expression of sinusoidal VIM and fewer Kupffer cells (**Figure 3F**) compared with the CP group.

**Figure 4:**
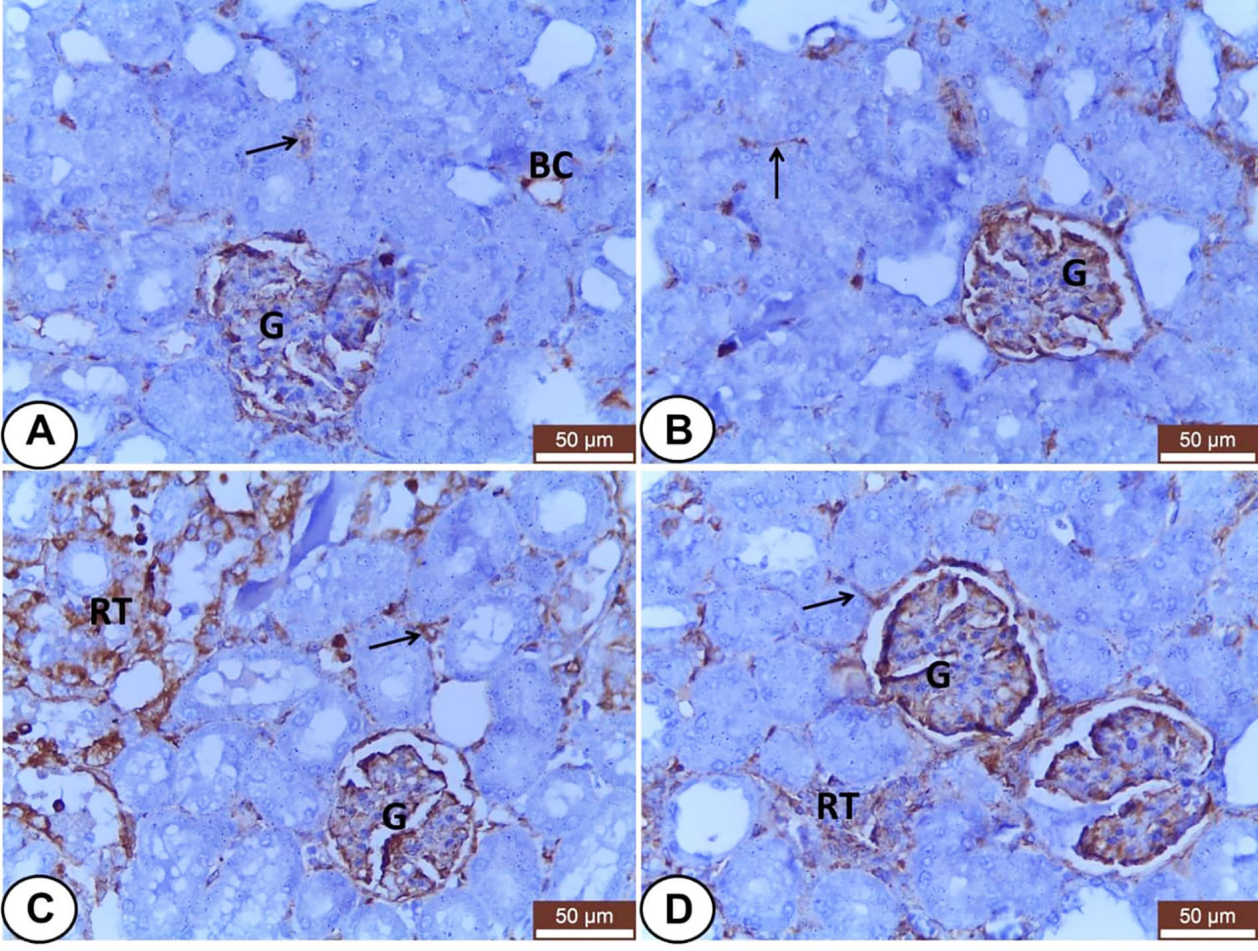
Immunohistochemical staining of VIM in renal sections from all examined groups. **(A, B)** Control and LC groups showed weak to moderate VIM staining in the interstitial tissues (thin arrow), blood capillaries (BC), and glomeruli (G). **(C)** CP group revealed moderate glomerular (G) and strong tubular (RT) and interstitial (thin arrow) VIM staining. **(D)** LC+CP groups showed less VIM reactivity in comparison to CP group especially the interstitium (thin arrow) and renal tubules (RT). Scale bars = 50 µm.

In kidneys, VIM was expressed mainly in the glomeruli, some peritubular blood capillaries, and interstitial fibroblasts of all experimental groups (**Figure 4**). In both control and LC groups, the renal tubules did not express VIM protein but, the interstitial tissues and glomeruli showed weak and moderate VIM staining, respectively (**Figures 4A, B**). Otherwise, overexpression of VIM was seen in the damaged renal tubules and interstitial tissues of the CP treated group (**Figure 4C**) that tended to be decreased in the interstitium and renal tubules of LC+CP group compared with CP group (**Figure 4D**).

### CK18 Expression

**Figure 5:**
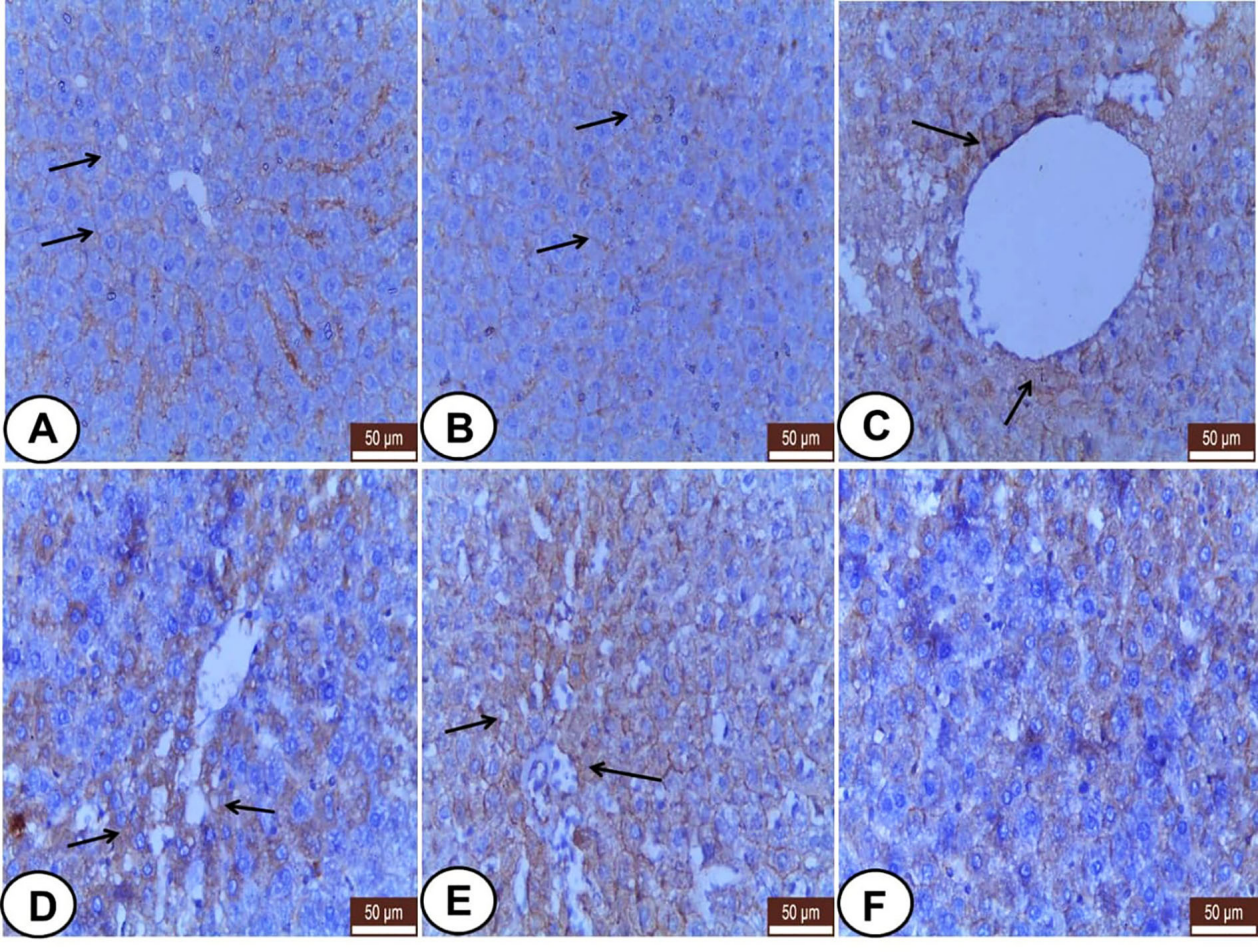
Immunohistochemical staining of CK18 in hepatic sections from all examined groups. **(A, B)** Control and LC groups showed weak CK18 staining at the periphery of the hepatocytes in reticular pattern (thin arrows). **(C–E)** CP group revealed strong, dense, and clumped CK18 staining in hepatocytes surrounding the central veins (thin arrows), fat cells (thin arrows), and triad area (thin arrows). **(F)** LC+CP group showed less CK18 staining compared with CP group. Scale bars = 50 µm.

Hepatocytes of both control and LC rats showed weak CK18 staining at the periphery of the cells giving reticular appearance (**Figures 5A, B**). CP group revealed strong, dense, and clumped CK18 staining in the hepatocytes surrounding the central veins, fat cells, and triad area (**Figures 5C–E**). In addition, the LC+CP group showed lower CK18 immunostaining compared with the CP group (**Figure 5F**).

**Figure 6:**
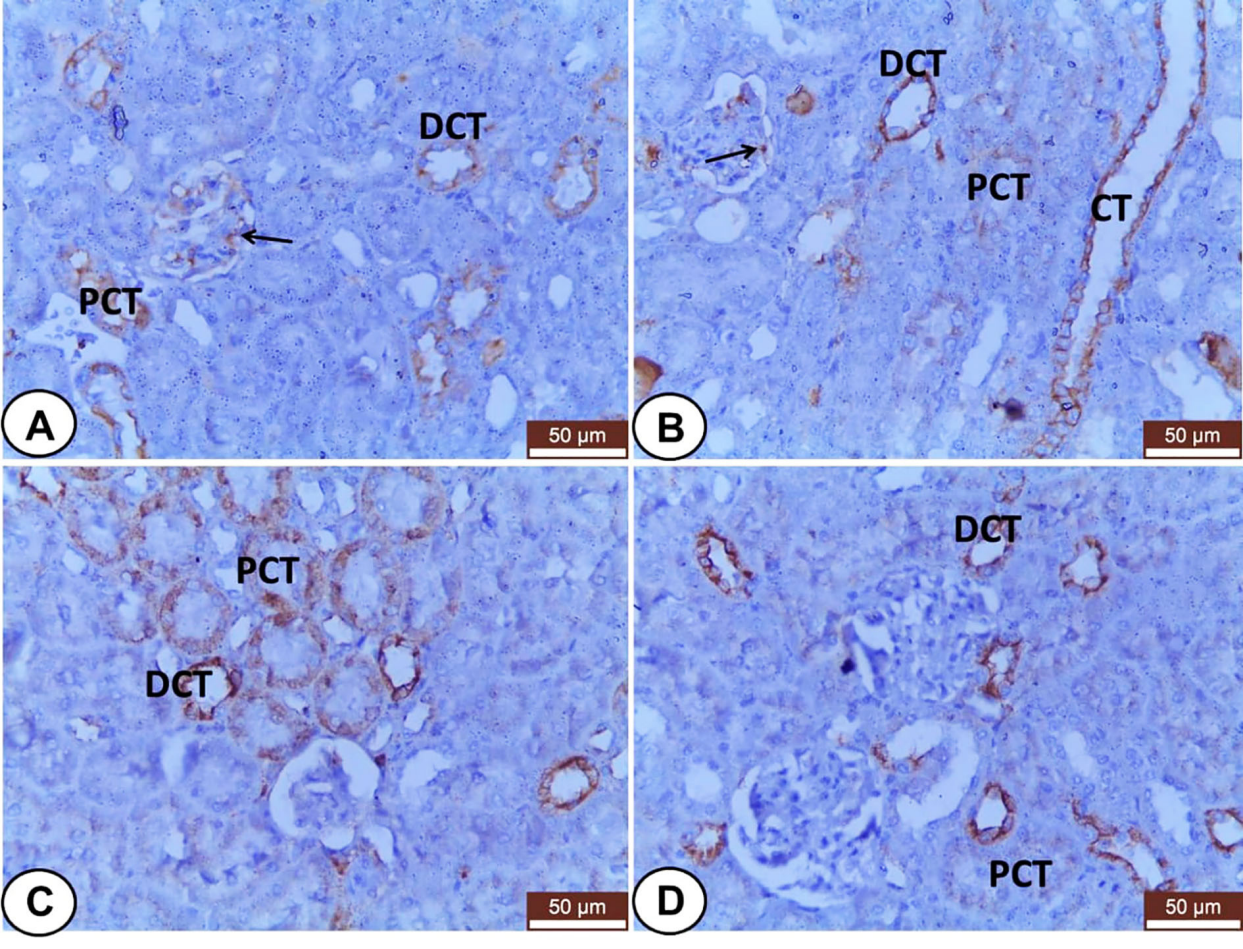
Immunohistochemical staining of CK18 in renal sections from all examined groups. **(A, B)** Control and LC groups showed weak to moderate cytoplasmic immunostainings in the visceral cells layer of Bowman’s capsule (thin arrow) as well as the cells of different segments of renal tubules; proximal convoluted (PCT), distal convoluted (DCT), and collecting (CT) tubules. **(C)** CP group revealed overexpression of CK18 in the epithelial cells of renal tubules mainly proximal convoluted (PCT) and few distal convoluted (DCT) tubules. **(D)** LC+CP group displayed lower expression of CK18 in PCT and DCT compared with CP group. Scale bars = 50 µm.

Regard to kidneys, both control and LC groups demonstrated weak to moderate CK18 immunostainings in the visceral cells layer of Bowman’s capsule as well as the cells of different segments of renal tubules; PCT, DCT, and CT (**Figures 6A, B**). However, CK18 was overexpressed in the epithelial cells of renal tubules mainly PCT and few DCT after injection of CP (**Figure 6C**). Meanwhile, the LC+CP group showed a lower expression of CK18 (**Figure 6D**) compared with the CP group.

## Discussion

Hepatic and renal toxicity is the most common dose-limiting side effects of CP-induced chemotherapy (Neamatallah et al., 2018). Reducing the potential side effects of CP by LC can be helpful during chemotherapy. Elevated activities of liver enzymes are known to be markers of cellular leakage and loss of functional integrity of hepatocytes because they are released into the bloodstream when the hepatocyte plasma membrane is impaired (Jia et al., 2018; Farid et al., 2019; Fadl et al., 2020). In this work, CP-induced hepatotoxicity was evidenced by significant alternation in serum liver enzymes (AST, ALT, and ALP). This result may be attributed to the metabolism of CP. Mohamed and Badawy (2019) reported CP is significantly taken up by the liver and accumulated in the hepatocyte, causing its damage leading to an increase of the liver enzymes activities. In addition, CP elevates creatinine and urea levels, which are confirmed by Sadeghi et al. (2020) and indicate nephrotoxicity induced by CP. On the other hand, Cayir et al. (2009) attributed the hepatotoxicity and nephrotoxicity induced by CP to the free radical generation in the renal and hepatic cells results in lipid peroxidation and oxidative stress that responsible for cellular damage.

Cisplatin administration produced a significant decrease in the total proteins and albumin, this result is supported by the result of Abuzinadah and Ahmad (2020). The above-mentioned results indicate disturbances in protein metabolism induced by CP intoxication due to a reduction in protein synthesis, following liver damage and alteration of functional integrity in the kidney leading to proteinuria, so, their plasma level decreases in hepatotoxic/nephrotoxic conditions (Sen et al., 2013).

Concerning oxidative stress/antioxidant parameters, the results of these parameters in the CP treated group is compatible with El-Shitany and Eid (2017) and Abd El-Kader and Taha, 2020, and Abdel-Razek et al. (2020) in the liver and kidney tissues, respectively. These results own to oxidative stress that is mediated through the generation of reactive oxygen species (ROS) such as superoxide anion and hydroxyl radical and depletion in plasma antioxidant levels (Fadl et al., 2019; Abuzinadah and Ahmad, 2020). So, our results are in harmony with several experimental and clinical studies suggesting that the oxidative stress through the formation of free radicals is one of the mechanisms of CP-induced hepato-renal toxicity (Ibrahim et al., 2018; Neamatallah et al., 2018; Abuzinadah and Ahmad, 2020).

On the other side, LC is a natural nutrient and necessary for the oxidation of fatty acid in the mitochondria to produce ATP (Tunez et al., 2007). So, it has antioxidant properties and plays protective roles against oxidative stress in various tissues, including liver and kidney (Cayir et al., 2009). In this study, the result of the LC+CP treated group is in accordance with Tunez et al. (2007) and Aboubakr et al. (2020), who reported that LC reduces liver enzyme activity, oxidative stress, and damage caused by thioacetamide and tilmicosin in rats. Also, LC can improve the antioxidant enzyme activity, including CAT and GSH, and reduces the MDA concentration in renal tissues in acute renal failure induced by myoglobinuric in rats (Aydogdu et al., 2006). These results were confirmed by the results of histopathology. The ability of LC to significantly improve liver and kidney biochemical parameters may be due to its antioxidant effect and its capability to act as a free radical scavenger, leading to the protection of membrane permeability (Augustyniak and Skrzydlewska, 2009). LC prevents oxidative stress and exerts a protective role against mitochondrial toxic agents (Barhwal et al., 2007). Additionally, they reduce the harmful effects of free fatty acids by enabling β-oxidation (Furuno et al., 2001).

The histological and immunohistochemical observations of the current study are in harmony and confirmed the alterations of the biochemical and oxidant/antioxidant parameters among the experimental groups. Several histological changes in the liver among CP treated group were noted as recently mentioned by Abuzinadah and Ahmad (2020). The central vein and sinusoidal dilatation and congestion are in accordance with Cagin et al. (2015) and Omar et al. (2016). Also, swelling of hepatocytes, hydropic degeneration, and prominence of Kupffer cells are demonstrated in similarity to El-Shitany and Eid (2017) and Neamatallah et al. (2018). In addition, fatty infiltration with signet ring appearance in some hepatocytes was seen as noted by Omar et al. (2016) and El-Shitany and Eid (2017). Otherwise, the pretreatment of CP treated rats with LC can potentially protect the liver against CP-induced histological changes and significantly improve and normalize liver histology that represents by almost normal hepatocytes and sinusoids, but with mild congested central vein with regression of the fatty changes. These findings are similar to El-Shitany and Eid (2017), indicating that LC can attenuate the hepatotoxic effect of CP (Cayir et al., 2009).

Like the recent finding of Abd El-Kader and Taha (2020); Abdel-Razek et al. (2020) and Sadeghi et al. (2020), the current study exhibited many distinguishing degenerative changes in the kidney of the CP group including degeneration and desquamation of the tubular epithelium, congestion, and dilatation of interstitial blood vessels and capillaries as reported by Neamatallah et al. (2018). Moreover, the presence of eosinophilic hyaline casts in some renal tubules agrees with Abd El-Kader and Taha (2020) and Abdel-Razek et al. (2020). Additionally, the deformity of some glomeruli with a widening of glomerular spaces was detected in accordance with Abd El-Kader and Taha (2020). On the other hand, LC administration ameliorated the histological effects of CP on the kidney, but with mild histological findings evidenced by tubular injury in some renal tubules, and minimal interstitial congestion. This finding confirmed that LC could attenuate the nephrotoxic effect of CP (Yürekli et al., 2011).

The current study focused on immunohistochemical localization of VIM and CK18 in hepato-renal specimens since VIM and CK18 expressions in the liver and kidney had provided a valuable insight into their microanatomy in both healthy and diseased conditions. A co-expression of VIM and CK in areas of damaged tissues was reported (Moll et al., 1991; Stefanovic et al., 1996).

Concerning VIM protein, it has been linked with several pathophysiological conditions such as cancer, rheumatoid arthritis, and HIV (Danielsson et al., 2018).

The detectable VIM in hepatic sinusoids of the present study confirmed the report of Evans (1998) where VIM is widely expressed IF proteins in endothelial cells and fibroblasts. According to Wang et al. (2017), VIM expression in the hepatic sinusoids may reflect its regulatory role of hepatic sinusoidal flow. At the beginning of this decade, both Golbar et al. (2011) and Aiad et al. (2012) regarded the sinusoidal VIM expression as hepatic stellate cells (HSCs), which are normally localized at the space of Disse. Moreover, Kupffer cells in the current study were VIM positive as mentioned by Sharifi et al. (2000) and Golbar et al. (2011). After the injection of CP, an overexpression of VIM in the liver was demonstrated in similarity to the lipopolysaccharide intoxicated liver (Lee et al., 2014). The numbers of VIM positive Kupffer cells were increased (Golbar et al., 2011; Aiad et al., 2012), and some individual hepatocytes became VIM positive (Aiad et al., 2012). As well as, sinusoidal VIM expression was increased in response to CP toxicity where HSCs are activated into myofibroblasts, which characterize by higher VIM expression and more secretion of the extracellular matrix so, they consider as the major contributor to hepatic fibrosis (Shang et al., 2018).

In the normal and diseased kidney, VIM is abundantly present in glomerular mesangial and epithelial cells (Gonlusen et al., 2001; Matos et al., 2007). Our study revealed VIM expression in the glomeruli of all examined rats confirming the above-mentioned data. In addition, VIM was expressed in the interstitial blood vessels and fibroblasts of all examined rats that agree with Stefanovic et al. (1996) and Sen et al. (2010) suggesting that the pivotal regulating role of the renal interstitium for vessels-tubules interaction as well as its involvement in the etiology of renal pathologies (Becker and Hewitson, 1997). It was interesting that the current results revealed undetectable VIM in the renal tubular epithelium of the control rats that agree with Skinnider et al. (2005) and Sen et al. (2010). Otherwise, the overexpression of VIM in renal tubules and interstitial tissue of the CP group was noted may be attributed to glomerulonephritis or tubule-interstitial injury (Gonlusen et al., 2001; Matos et al., 2007), respectively.

Cytokeratins of hepato-renal tissues increase in response to toxicants, oxidative stress, inflammation, and other damaging insults (Toivola et al., 2010). Hepatocytes, as one of the epithelial cells of the liver, have been known to express CKs (Ku et al., 2007). The hepatocytes of both control and LC treated rats showed faint CK18 at the cell periphery forming reticular staining patterns that agree with Zatloukal et al. (2004). Upon injury by CP, the intoxicated hepatocytes exhibited strong, dense, and clumped CK18 staining that agrees with Omary and Coulumbe (2004). Moreover, immunohistochemical overexpression of CK18 in hepatocellular carcinoma was noted (Sawan, 2009). These findings point to the structural role of CK18 to hepatocytes providing them mechanical stability. Another role of CK18 is a target and modulator of toxic stress (Zatloukal et al., 2004) therefore; the current study reported that the CK18 expression was significantly upregulated by oxidative stress induced by CP. That confirmed CK-18 levels as a predictor for hepatitis progression (Yang et al., 2015).

Cytokeratins are often used as disease markers in renal pathology and experimental research (Djudjaj et al., 2016). Expression of CK18 in the parietal cells of Bowman’s capsule as well as the different segments of renal tubules of the normal rats agree with the finding of Stefanovic et al. (1996) and Djudjaj et al. (2016). CK18 staining was stronger in the collecting ducts as mentioned by Sen et al. (2010). Like Snider (2016), our finding showed a pronounced overexpression of CK18 in the damaged renal tubules in CP treated group indicating that CK18 was upregulated by tubular injury. Therefore, CK18 can be used as a marker and regulator of renal tubular epithelial injury (Djudjaj et al., 2016).

It was noteworthy to record that the pretreatment of LC could decrease expressions of VIM and CK18 in hepato-renal tissues compared with CP group indicating the ameliorative role of LC against CP toxicity, especially in restoring the organization of IFs.

## Conclusions

Cisplatin induced hepato-renal toxicity associated with oxidative damage, lipid peroxidation, histological changes, and disorganization of the cytoskeleton IFs; VIM and CK18. A daily LC treatment at 100 mg/kg exerts ameliorative effects against CP-induced hepato-renal toxicity. The antioxidant effect of LC was evidenced by the restoration of activities of oxidative/antioxidant markers, histological pictures, and VIM and CK18 organization in hepato-renal tissues. Therefore, this study suggests LC as a supplement for cancer patients under CP treatment.

## Data Availability Statement

The raw data supporting the conclusions of this article will be made available by the authors, without undue reservation.

## Ethics Statement

The animal study was reviewed and approved by Faculty of Veterinary Medicine, Benha University, Egypt.

## Author Contributions

All authors contributed equally to this article. SA and MA-D funding acquisition and project administration and assisted in writing and editing the manuscript with other authors.

## Funding

This work was funded by Researchers Supporting Project number (RSP 2020/27), King Saud University, Riyadh, Saudi Arabia.

## Conflict of Interest

The authors declare that the research was conducted in the absence of any commercial or financial relationships that could be construed as a potential conflict of interest.
